# A Qualitative Investigation of Music Use among Amateur and Semi-Professional Golfers

**DOI:** 10.3390/sports7030060

**Published:** 2019-03-05

**Authors:** Nicole T. Gabana, Jasmin Hutchinson, James Beauchemin, Matthew Powless, Julia Cawthra, Aaron Halterman, Jesse Steinfeldt

**Affiliations:** 1 Department of Educational Psychology and Learning Systems, Florida State University, Tallahassee, FL 32306, USA; 2 Department of Exercise Science, Springfield College, Springfield, MA 01109, USA; jhutchinson@springfieldcollege.edu; 3 School of Social Work, Boise State University, Boise, ID 83725, USA; jamesbeauchemin@boisestate.edu; 4 Department of Counseling and Educational Psychology, Indiana University, Bloomington, IN 47405, USA; mpowless@indiana.edu (M.P.); cawthrajulia@gmail.com (J.C.); awhalterman@gmail.com (A.H.); jesstein@indiana.edu (J.S.)

**Keywords:** music, golf, tempo, attention focus, mood regulation

## Abstract

Music use in golf receives minimal attention from both applied and empirical perspectives. Golfers, coaches, and sport psychology practitioners alike may benefit from understanding and utilizing music within their work. Since music use in golf has become an increasingly common practice, the purpose of the current study was to investigate current music use among golfers using a qualitative approach. Researchers aimed to identify potential psychological and physiological effects derived from music use during golf practice and pre-performance, given the limited empirical research in this area to date. Semi-structured interviews were conducted with ten amateur and semi-professional golfers (five male, five female, *M_age_* = 22.9 years, *SD* = 5.04 years). Consensual qualitative research (CQR) methodology was used to analyze the interview data. Six domains emerged from the CQR analysis regarding participants’ self-reported music use in golf: tempo, attention, physiological regulation, psychological regulation, effects of music on performance perceptions, and context (to use or not to use). Given the capacity of carefully selected music to elicit profound affective, neurophysiological, and behavioral responses, there is clear potential for mental performance consultants to utilize music in working with golfers in training contexts. Implications, caveats, and future research recommendations are provided.

## 1. Introduction

Over the past two decades, there has been a proliferation in empirical research on the effects of music in sport and exercise settings, although the use of music in golf remains understudied relative to music use in other sports. Music is highly utilized among both amateur and professional athletes, especially during pre-performance preparation. Many prolific sport stars such as Usain Bolt, Rafael Nadal, and Michael Phelps can be observed wearing headphones before competing, and have testified to the importance of music within their pre-performance routine. Athletes have demonstrated a range of interpretations of how music can affect both pre-competition preparation and subsequent performance [1]; however, little is known about how or why golfers use music. This is particularly pertinent since golf primarily requires discrete, fine motor skills, whereas the majority of empirical studies focused on continuous gross-motor activities such as running, cycling, swimming, and rowing. To date, the authors are aware of only one study that examined the effects of music on golf performance. 

Baghurst, Tapps, Boolani, Jacobson, and Gill [2] investigated the effects of different musical genres (jazz, classical, country, rock, and hip-hop/rap music) on putting accuracy in experienced collegiate golfers. With the exception of rock music, participants performed significantly better on the putting task in all musical trials compared to a no-music condition. Furthermore, participants were found to perform significantly better while listening to jazz compared to other musical genres. While this study is the first known empirical investigation of music on golf performance, listening to music while training (e.g., putting, driving) is a common practice among golfers, especially millennials. Following the development of mp3 players (e.g., iPods), the emergence of smartphones has made listening to music increasingly more portable, affordable, and common. This increased accessibility has made it easier than ever to utilize music in sport, a trend present in both athletes and exercisers alike [3]. Amateur, semi-professional, and professional golfers are among those who have been observed listening to music during both training and pre-performance; therefore, more research is needed to explore how golfers use music, which is the focus of the current study.

The benefits of music listening in a sport setting are numerous, and each has received strong empirical support. An overview of relevant research findings follows.

### 1.1. Optimizing Arousal and Affective Valence 

Music can be used as either a sedative or a stimulant to engender the optimal arousal state prior to and during a competition. Research broadly supports the assumption that stimulative music increases psychomotor arousal, while soft or sedative music decreases arousal and facilitates relaxation. Several studies have shown increased activation of the sympathetic nervous system with fast-tempo music [4]. Conversely, listening to sedative music can lead to decreased heart rate, respiration rate, and blood pressure, indicating an increase in parasympathetic activity [5]. Music may also influence arousal if it evokes an extra-musical association that either inspires physical activity or promotes relaxation [6]. 

Mood and feeling states during training exercise tend to be more positive under music compared to no-music conditions [7]. Researchers have attempted to tease out which particular aspects of music influence emotional states in listeners. Two key elements are tempo and mode (i.e., major or minor key). Music generally classified as “happy” sounding is characterized by fast tempo and major mode, whereas music interpreted as “sad” sounding is typically played in slow tempo and minor mode [8]. While fast tempo music has been associated with enhanced athletic performance, golf may warrant different musical qualities for optimal results. Aiming and shooting sports, such as golf, require a relatively low level of physiological arousal coupled with a low level of cognitive anxiety [1]. Slower tempo music has been associated with decreased psychological and physiological arousal, as well as reduced anxiety [9,10]; however, more research is needed to explore the types and perceived functions of music use among golfers who are listening to music in real-world settings. 

### 1.2. Synchronization

Humans have an innate tendency to synchronize movement with rhythms. One obvious example of this is our natural tendency to tap our feet when listening to music [11], or to synchronize our walking or running stride with a musical beat [12]. The synchronous application of music in a sport setting has an ergogenic (work-enhancing) effect on performance [13] and is associated with better running economy [7]. Timing is critical in the generation of coordinated motor actions [14]. Indeed, at its essence, a golf swing is the result of a complex sequence of precisely timed muscle contractions. Timing and rhythm go hand in hand, yet there is limited systematic research on the use of music in sport skill learning and performance. This is despite findings supporting the positive effect of rhythmic accompaniment in other areas, such as the development of fundamental motor skills in young children [15] and gait training in stroke patients [16]. Synchronized metronome training (SMT) has been used in a variety of sports settings, including tennis and golf. Sommer and Rönnqvist [17] found that experienced male golfers who participated in four weeks of SMT significantly improved their golf shot accuracy while a control group did not. A follow-up study indicated that improved motor timing, as an effect of SMT, led to a more coordinated and dynamic swing performance, with decreased variability in the temporal structure of the swing motion. 

Sonification of movement, which involves digitally transforming human movement data into sound, emerged in the motor skill learning literature as an effective method of providing concurrent augmented feedback [18]. For example, motor learning in an indoor rowing task was significantly enhanced by means of real-time movement sonification, when compared to visual feedback and to audiovisual feedback with natural movement-attended sounds [19]. Sonification of movement might be advantageous to metronome training as it can also have a motivational effect and shows potential for use in movement error detection [20]. Athletes also appear to prefer the esthetic qualities of music or natural sounds over pure tone [21]. Given the natural synchronization effects of music, it is possible that music might elicit performance benefits in golf with regard to swing timing and tempo. 

### 1.3. Attentional Focus

Attention refers to the allocation of limited-capacity mental resources to processing [22]. In a sport setting, and particularly in golf, there is considerable evidence that directing attention externally to the effect of a movement (e.g., focusing on the trajectory of the golf ball) improves performance compared to directing attention internally (e.g., toward bodily movements involved in the execution of the golf swing [23,24]). Music is considered to be a distractive stimulus that can facilitate external attentional focus by occupying limited channel processing capacity, thereby distracting the listener from internal sensations [25]. Although the vast majority of research findings support this hypothesis, qualitative data suggest considerable variability in how people use music during exercise [26]. Thus, a more nuanced approach to the use of music in sport is warranted, wherein music may promote both an internal and external focus of attention depending on task characteristics [26] and/or individual attentional and motivational preferences [27]. Moreover, the use of music to block external distractions may simultaneously promote sustained attention on the task at hand. Here, athletes “use music to block out distractions in the competitive environment, form their own listening bubbles, and create an optimal mindset” [1] (p. 173).

## 2. Purpose of the Study and Research Questions

The purpose of the study was to gain a better understanding of music use among golfers using a semi-structured interview guide derived from an empirically supported framework of the effects of music on sport performance. The subjective nature of music perception and preference appears to require subtle methods of investigation [28]. Within the domain of music research in the sport and exercise sciences, qualitative research that adopted the methodology of open-ended interviews [29,30] proved particularly effective in elucidating the subtle relationships which exist “in a field of study that inevitably revolves around individual perceptions” [30] (p. 349). The current study aimed to address two research questions:

1. How are golfers currently using music?
2. Based on golfers’ reported perceptions and experiences of music use, as well as previous research on the effects of music on sport performance, what are the potential benefits of music use in golf?

## 3. Materials and Methods

### 3.1. Participants

Ten golfers (five male, five female) between the ages of 18 and 34 years old (*M*_age_ = 22.9 years, *SD*_age_ = 5.04 years) were included in the study. Participation was based on the following criteria: adult golfers who reported using music regularly (i.e., weekly) during practice and/or pre-performance. Three participants self-identified as Asian and seven identified as Caucasian. Participants’ experience playing golf ranged from 4 to 22 years, with a mean experience of 10.3 years. Nine of the 10 participants had handicaps ranging from 2 to 14 (*M* = 5.44); one participant reported that she did not use a handicap. 

### 3.2. Procedure

Golfers were recruited through convenience sampling via the primary researcher’s proximity to golfers at the amateur and semi-professional levels. After participants provided informed consent, semi-structured interviews were conducted in-person by the primary researcher with all 10 participants. Interviews were then transcribed verbatim and member-checked by participants for accuracy. All participants confirmed that their original responses accurately reflected their attitudes and experiences related to their music use in golf. After data collection was complete, a qualitative research team was assembled to analyze the participant responses. The development of the interview protocol and data analysis procedures are outlined in the following sections.

### 3.3. Interview Protocol

Given that, to our knowledge, only one study to date examined music use in golf [2], literature in the general area of music use in sport was reviewed. Interview questions were generated using a “start list” derived from common empirical themes in the existing literature, with guidance from an external expert researcher. These questions related to performance, music choice, attention, affect, effort, tempo, skill acquisition, and imagery. Consistent with recommendations by Hill [31], additional probes were considered in advance; the interviewer also created spontaneous probes during the semi-structured interview process to gather a breadth and depth of information. The interview guide was pilot-tested with a golfer who was not included in the study [32]. 

### 3.4. Data Analysis

Consensual qualitative research (CQR) [31] was the qualitative methodology used in this study. The CQR method incorporates elements of several qualitative approaches (e.g., phenomenological, grounded theory) and integrates open-ended questions, multiple researcher perspectives, consensus data interpretation, the use of an auditor, and domains, core ideas, and cross-analyses [31]. 

The primary research team consisted of two 27-year-old Caucasian females, a 27-year-old Caucasian male, and a 32-year-old Caucasian male. A tenured faculty member from the field of sport and exercise psychology who was not a member of the primary research team provided guidance as an external expert researcher. Another tenured faculty member with expertise in sport psychology and CQR was recruited for the role of external auditor [32]. Prior to data analysis, the primary research team met to discuss biases. Members of the research team identified as former college athletes (football, rowing, volleyball, wrestling) but none were collegiate golfers. Assumptions about golf and golfers, as well as the use of music in sport, were discussed. Researcher biases were continually checked throughout the CQR process to increase transparency and awareness amongst the research team [31]. 

Team members were trained in CQR methodology and discussed any questions or concerns with the primary researcher. The four members of the primary research team read each of the transcriptions individually to become fully immersed in the data. After independently coding the data, the research team met on multiple occasions to reach consensus on emergent domains [31]. Next, research team members returned to the transcripts to abstract core ideas from participant responses. Core ideas represent common phrases that remain as close to participant wording as possible, in order to reduce misinterpretations, assumptions, and redundancy [32]. Two researchers reread each transcription individually to review core ideas and domains, serving as internal auditors during the case analysis process. The team then met to assign core ideas to respective domains, moving through multiple iterations and making revisions when agreed upon by the team. 

The next step consisted of a cross-analysis procedure, in which the research team developed categories within domains across cases [31]. The researchers intentionally returned to the raw data frequently, to ensure accurate placement of categories and domains. Discrepancies were discussed and codes revised based on mutual agreement. At this point, the external auditor reviewed the cross-analysis and provided feedback on the generated domains, core ideas, and categories. A benefit of having an external auditor is that “they can provide a perspective on the data that is not as influenced by groupthink” [32] (p. 201). Several revisions were made based on feedback from the external auditor and a cross-analysis was conducted to determine the final categorization structure.

## 4. Results

Semi-structured interviews and CQR analysis indicated a multifaceted functionality of music use among golfers. Six domains emerged: (1) tempo, (2) attention, (3) physiological regulation, (4) psychological regulation, (5) effects of music on performance perceptions, and (6) to use or not to use.

Firstly, findings regarding music preferences among participants are summarized. Then, the domains and categories are presented, organized by general responses (endorsed by all, or all but one; 9–10 participants), typical responses (endorsed by more than half; 6–8 participants), and variant responses (endorsed by less than half, but at least two; 2–5 participants). Domains and categories are described and illustrated using core ideas and participant quotations when appropriate. General and typical responses are reported in the results section, while all responses are outlined in Table S1 (Supplementary Materials). 

### 4.1. Music Preference

Participants reported that they started using music anywhere from 2 to 10 years prior to the time of the interview (*M* = 6 years). All 10 golfers reported using music regularly during practice and/or pre-performance. Of the nine golfers who were currently competing at the time of data collection, five reported using music every day, while four reported using music 50% of the time or approximately 2–3 days per week. One participant who was not currently competing at the time of the interview said she used music regularly during her collegiate career, but reported current “occasional” music use since she does not play as often. She was asked to respond based on her knowledge and experience of using music regularly during her competitive career, which ended approximately 1 year prior. While golfers differed slightly in their frequency of music use, all participants were considered to be regular listeners of music in golf for at least 2 years at the time of data collection, granting them inclusion in the study. When asked their preferred genre for golf music, participants varied in their responses, with some golfers endorsing more than one genre. Genres identified by participants included alternative/indie rock (*n* = 2), country (*n* = 4), hip-hop (*n* = 2), instrumental (*n* = 3; e.g., classical, acoustic), international (e.g., Asian artists/bands; *n* = 3), oldies (*n* = 2), rap (*n* = 2), rhythm and blues (R&B; *n* = 3), reggae (*n* = 1), rock (*n* = 2), soft rock (*n* = 3), techno (*n* = 1), top 40 hits/pop (*n* = 2), and worship (*n* = 1). 

### 4.2. Tempo

Participants identified a connection between the function of their music use in golf and musical tempo or beat. Two categories, mental synchronization (general) and physical synchronization (typical) effects emerged from the findings.

**Mental synchronization**. Core ideas identified within this general category included the following: the beat is helpful, wrong tempo can disrupt performance, and playing music in one’s head when not listening to it can be helpful. In general, participants indicated a preference for a slow-to-moderate tempo music when playing golf. One participant indicated the following:

“I would listen to a slower rhythm no matter what. I wouldn’t listen to something crazy fast and upbeat, it would have to be slower.”

When asked why, he said,

“That’s the way, uh, your mind has to work in golf. The faster you’re going the, I think the less you stay focused. And you, you’re constantly trying to slow down in golf. Slow down everything. Slow down the way your mind thinks, think about less … [music] takes away the conscious element of what I’m actually doing … I’m not thinking about what I’m doing, just thinking about the rhythm.”

**Physical synchronization**. Physical (i.e., auditory–motor) synchronization with the musical beat also emerged as a typical category. The core ideas grouped within this category were choosing music for the purpose of timing one’s swing, comparing music to a metronome, and synchronizing one’s swing to the tempo of the music. A participant explained how he selected music (e.g., Frank Sinatra) to match his tempo in golf.

“I heard it and it, well ya know, it just seems to work. Once you hear a song and you feel the melody or rhythm then you know you have to, well you just associate it with golf automatically. Just because of the pace that it’s played. You wouldn’t, you wouldn’t ever run and play golf at the same time, so you wouldn’t listen to something fast-paced and play golf at the same time … You wouldn’t want your heart racing like you would have it at a rock concert.”

### 4.3. Attention

Participants identified music as facilitating both an associative and dissociative attentional focus in relation to performance, constituting two categories within this domain. Responses indicated that music can help narrow focus to helpful thoughts about the task at hand, while simultaneously functioning as a distraction from unhelpful thoughts or other people, working as both an associative and dissociative mechanism (congruent with findings of Gabana et al. [27]). Furthermore, internal and external themes were prevalent, consistent with Wulf’s [23,24] framework. Music can also serve as a memory cue for pleasant golf-related or golf-unrelated memories (variant).

**Music functions as an associative mechanism**. The core ideas expressed in this general category were that music functions as a focus mechanism, using music to concentrate on task, and to narrow one’s focus to helpful thoughts. Illustrating this function, a participant commented,

“I need to just stay relaxed and focus on getting it on the fairway, getting it on the green, it doesn’t matter whether you’re far away from the pin, just as long as you’re in a good position then you’re able to score … so, the music helps me to just think of that.”

**Music functions as a dissociative mechanism**. Participants also described using music to dissociate from unhelpful thoughts or distractions (e.g., other people), which constituted the two core ideas in this category. A participant explained how music affected her swing thoughts:

“It somewhat distracts me from all the different things that I’m trying to do in the golf swing, and it simplifies my thoughts I think … it helps me not think about my swing so much.”

Another participant spoke of using music to facilitate optimal focus and automaticity in performance:

“Why do I think it’s helpful … to focus more on the music than it is to focus on golf? Um, ‘cause the last thing I wanna do is focus on golf … it’s always on your mind, but if you’re actually doing it, it helps if you’re not thinking about it at all … it would be like somebody thinking about reading when they’re reading. Does that make sense? Would you think about how well you’re reading? Or what you need to do in order to read the words? Or would you just go ahead and read the words? Or anything that you do enough, you don’t wanna think about it.”

### 4.4. Physiological Regulation

The third emergent domain focused on how music use related to physiological sensations during golf. Within this domain, only one category qualified as a general theme.

**Music helps to regulate energy/arousal level**. Overall, participants reported that music helped them relax physically (e.g., calming nerves), as well as increasing energy if they felt too sluggish. Another core idea within this category was using music to help regulate physical/physiological feelings such as tension in the body. Many golfers reported choosing music specific to their desired physiological state for optimal golf performance. For example, one participant explained,

“If I’m like feelin’ a little tired or not feelin’ really into practicing I’ll listen to a little bit more upbeat, and then after sorta like, woken myself up a little bit and got back into practicing then I’ll put something a little bit calming in so I can just really get in, focused.”

Another noted the calming effects of music on her nerves:

“If I just talk to someone, I don’t think that’s helpful… they always like, ‘oh don’t be nervous just relax’ but when they say that you will be like, nervous. So, I just listen to music, because like the music’s not gonna say that [laughs] … I always like listen to music like in walk alone before I tee, tee off. I don’t want like someone with me like before or if someone talks to me like, ‘okay just play your game’ or something like that. That’s gonna be like, make me nervous.”

### 4.5. Psychological Regulation

Participants identified the value of music in regulating mood and other psychological aspects of performance. Two general categories (music helps to regulate mood, and music enhances mental performance state) and one typical category (music increases motivation) emerged from the data.

**Music helps to regulate mood**. Four core ideas were drawn from the data in this category. Participants reported using music as a preventative mechanism from becoming too angry/frustrated, a method to put oneself in a better mood, choosing music based on one’s mood, and the ability of music to change one’s mood. One participant stated,

“It keeps me like, just happy though, like if I get upset with myself when my swing sucks or something, just listen to music, it just fixes everything.”

Another commented on how listening to music helped him maintain a balanced mood state:

“The bad doesn’t seem so bad and the good probably doesn’t seem so good because you just get into a rhythm and zone everything out. So you’re not thinking about the results so you, you don’t really get caught up in, the emotions just kinda stay the same”

Interestingly, another participant spoke about choosing music to validate her current mood:

“If I’m like upset or angry about how I’ve played I’ll listen to like, not necessarily angry music, but ya know something a little bit louder. It helps me feel like it’s okay.”

**Music enhances mental performance state**. In general, participants frequently commented on how music can put them in a better psychological/mental state to play golf (e.g., mental preparation), help them recover from poor performance, and increase their confidence. When asked about how she uses music, a participant remarked that music helps her to stay in the present:

“Just to keep me relaxed and not think so much about where I hit the ball or whenever after like, I hit the range or I’m done and I go for my tee time I just listen to music and just forget about everything that just happened.”

One participant explained how music can serve as a pathway to a desired mood state, purporting that confidence may be a potential mediator between music and enhanced performance:

“I pick it based on the mood that I’m feeling; so sometimes if I’m feeling a little down about things, I might play something that’s like an upbeat, so I’ll like, it can pick me up and I can build confidence. I think music builds confidence, and confidence will improve or enhance your game, um, but music doesn’t really directly correlate with your success … the music that helps you become more confident; therefore, you’re probably gonna play better if you’re more confident.”

Another spoke similarly of the impact of music on her confidence level:

“When I’m doing a practice round, I concentrate on the music and I realize I’m hitting it good, and it gives me a lot of confidence knowing that tomorrow I can play good. So what I do is I just sing that song in my head and it just helps me focus and know that I can play well.”

**Music increases motivation**. In the same vein as confidence, a typical theme was the use of music to increase motivation. In addition to enhancing general levels of motivation, some participants noted that the lyrics of a song specifically contributed to increased feelings of motivation, constituting a core idea. This is highlighted in the following participant’s depiction of persistence:

“Even if I start getting a little bit bored of it like the music is still like in the background so it’s like ‘alright so you’re gettin’ a little bit bored but you know what, like you’ve got some good music goin’, just like keep practicing.’ So, it’s kinda like a little bit motivational.”

### 4.6. Effects of Music on Performance Perceptions

Participants identified other ways that listening to music can affect experiences when practicing or performing. In this domain, two general categories (music affects time perception, and music functions as a performance enhancer and facilitates flow) and one typical category emerged (music improves the qualitative experience of the task).

**Music affects time perception**. Overall, golfers perceived that time went faster when listening to music. To illustrate this point, a participant stated,

“[Music] makes it more fun and can make time go by quicker. Like you can be out there hittin’ golf balls for two hours and then kinda have this song goin’ in your head and then ‘oh crap two hours has gone by’… if you’re just out there hittin’ balls you can kinda get sick of … yea I think it makes things easier.”

**Music functions as a performance enhancer and facilitates flow**. Within this category, five core ideas emerged from the data: greater efficiency when listening to music, the task seems easier when listening to music, music enhances performance, music increases effort level, and music facilitates flow state and gets me “in the zone”. One of the participants elaborated on this latter point:

“When I listen to music during practice, the music just gets me in the zone, ya know. Like it may be noise, but it just somehow enables me to focus and just get ready to play … since I played a lot of musical instruments, I have this thing where I start tapping away, like with my fingers and feet, so it makes me move faster, and makes me work more efficiently.”

When asked about the effects of music on performance, another participant explained:

“If you’re playing the same music that you practice with … you get more comfortable with your setting … you hit a hundred golf balls to this music, this song. So, the song can kind of soothe you, take away some of your nerves … I find myself throughout a round of golf I’ll have one song kind of stuck in my head … it helps me take away my surroundings.”

**Music improves the qualitative experience of the task**. This category was classified as typical, consisting of two core ideas: music makes golf more enjoyable and music prevents boredom. One participant summarized this sentiment in the following statement:

“It’s just me and like, me swinging the club, it just really simplifies it, like I’m just out there enjoying it … sometimes practice can be so like, repetitious … I think music has a positive influence on you and it can get you gaining your interest and just kind of get you more aware.”

### 4.7. To Use or Not to Use

In the final domain, common situations, settings, or conditions in which golfers deemed music use as helpful or unhelpful emerged from the data. Two general themes were found: social/individual and setting. Three typical categories related to music use in context were noted, pertaining to putting vs. driving, task difficulty, and using music in conjunction with imagery.

**Social/individual—music is used with other people and when practicing alone**. Participants referenced instances of listening to music both alone and with others. While individual participants differed in their preference for using music in social and/or individual settings, neither setting emerged as a primary use or preference in the interview sample. One participant indicated,

“During practice rounds are the best times for me to listen to music ‘cause like, we’ve played practice rounds with people who, who like to interact and most of them don’t really concentrate because not all are in tournament, so I’d rather use that time to take that advantage to get to know the course better and just excluding everybody out.”

In regard to solo use, a participant stated,

“Let me tell you, the only way I would play by myself was if I was listening to music.”

**Setting—listening to music is common during pre-performance routine and practice**. All participants reported using music while practicing golf, albeit at different frequencies. Most participants indicated using music as part of their pre-performance routine. When asked to elaborate on the purpose of listening to music before competition, one participant said,

“It gets me to start thinking about what I’m gonna have to do, and kind of just get my mind on the round that’s comin’ up.”

Another stated,

“Before like my competition … I always listen to like only like the most important music for me… make me feel like confident and like, get good attitude … before my like, tee off.”

**Difficulty—music may be unhelpful when learning new skills**. Many participants reported that if they were learning or practicing a new skill, they would not listen to music during this process. One participant explained,

“I think learning a new skill, it needs all your focus and attention, and so I don’t think music’s the best idea when learning a new skill right away, but after you’ve learned it.”

Another similarly explained,

“I’m tryin’ to think about what I’m doing, and I think the music kind of takes away from like thinking about movements. ‘Cause when I listen to music it’s more about the feel than it is about the mechanics.”

**Task—music is used more often in putting vs. driving**. While some endorsed using music on the driving range, participants consistently reported more frequent music use during putting practice. A participant explained how music increased his interest and effort on the putting green.

“Especially with short game, um which ‘cause you have to spend so much more time practicing with short game than anything else, um, I probably wouldn’t spend as much time if I was practicing. I think I would just get kinda bored.”

**Music is not commonly used in conjunction with imagery**. Given past research on music and imagery, a question was included to investigate whether golfers use music in conjunction with mental imagery (e.g., visualizing a target). While most reported using imagery frequently in their golf game, participants typically responded that they did not combine imagery with music use.

## 5. Discussion

The purpose of the study was to investigate music use among golfers and to begin to understand the potential effects of music use in golf. Research on music use in golf has been limited to one study on putting performance [2]; therefore, a qualitative approach was employed and a semi-structured interview guide was designed based on previous research on music use in sport, which has found numerous psychological, physiological, and performance benefits [1]. Results of the study indicated six major domains: (1) tempo, (2) attention, (3) physiological regulation, (4) psychological regulation, (5) effects of music on performance perceptions, and (6) to use or not to use. Given that the domains were explained individually in the results section, we focus on their integration in the discussion. 

Participants identified tempo as a key element in music selection and usage in golf, from both a mental and physical synchronization standpoint. Golfers preferred music with a slow-to-moderate tempo because of its ability to produce a calmer mood. All golfers reported that listening to music produced a calming effect, which enhanced their golf practice. This supports previous literature which found that listening to slower-tempo music can decrease blood pressure, heart rate, and respiration rate [5]. Additionally, Karageorghis [1] purported that golf requires a relatively low physiological arousal and low cognitive anxiety; therefore, golf may warrant slower-tempo music given its association with these effects [9,10]. While the theme of anxiety did not emerge in the current data, participants endorsed increased confidence and motivation when listening to music (particularly related to motivational lyrics). Additionally, some participants noted that, when practicing, they selected music to match their mood. Four participants associated certain songs with pleasant memories (both golf-related and golf-unrelated). This extra-musical association has been found to affect arousal if it promotes relaxation [6]. Thus, findings demonstrate connections among domains of tempo, physiological and psychological regulation, and attention.

Participants also discussed using music as a way to synchronize their swing with the beat of the song, echoing synchronization themes in other sport and exercise settings [33]. Multiple participants compared using music as they would a metronome—to time their swing with a particular beat in order to achieve a specific tempo conducive to optimal individual performance. This is similar to previous research which found that music listeners tend to synchronize their walking or running stride with the beat [12]. Since Professional Golf Association (PGA) rules prohibit using music during competition, some golfers said they would imagine a song playing in their head in order to maintain their desired tempo—a concept defined as auditory imagery [34]. Thus, imagining music may serve as both a mental and physical synchronization cue, even though participants reported they did not use music in conjunction with visual imagery practices. In other sports, synchronization demonstrated performance-enhancing effects [7,13]; therefore, more studies are needed to explore whether performance differences might be observed quantitatively for golfers who use heard or imagined music to synchronize their swing.

In regard to attention, golfers’ responses indicated use of music in both associative and dissociative capacities, in that music can serve as a distractive mechanism from unhelpful external or internal stimuli (e.g., chatter, excessive swing thoughts) and narrow focus to the task at hand. Participants noted that music can block out internal distractions, consistent with previous research which has found that music can facilitate an external attentional focus by occupying limited channel processing capacity [25]. According to the constrained action hypothesis, an internal attentional focus may “constrain or interfere with automatic control processes that would normally regulate the movement” [35] (p. 1143). Strategies to minimize an internal attentional focus may be particularly helpful in golf which involves fine motor skills, and where, at the amateur and professional levels, automaticity is key. It is possible that music facilitates this attentional shift, which may be related to the tempo and synchronization benefits of music reported by participants.

All participants stated that listening to music while golfing helped regulate both their energy/arousal and mood, and enhanced their overall mental performance state. Golfers explained that listening to music made them feel more mentally prepared to practice and compete, and helped them bounce back from poor performances. In addition, six golfers indicated that music made golf more enjoyable and decreased boredom when practicing, thus improving the qualitative experience of the task. Findings support the notion that mood and feeling states tend to be more positive under music conditions [7]. A caveat is that participants in the current study were selected based on their current use of music during golf; therefore, music use may not be appropriate or facilitative for all golfers. More research is needed to support these claims among the general population.

Other common sentiments shared by participants were that time seemed to pass more quickly when listening to music; the task seemed easier; they felt more efficient and effortful; their performance was better (e.g., putting); and they felt “in the zone”, resembling a flow state. Researchers have posited that music may facilitate the attainment of flow, which is linked to intrinsic motivation, increased efficiency, altered perception of time, and enhanced performance [36]. Participant responses also lent insight into the conditions in which music is most commonly used in golf. Overall, findings indicate that golfers listen to music individually (e.g., wearing headphones during independent practice), socially (e.g., playing music through a portable speaker at the driving range with friends), more frequently in putting than driving, and both during practice and pre-performance (e.g., on the way to a tournament). Eight participants reported that they do not tend to use music when learning a new task. While some shared the perception that listening to music would not be helpful during skill acquisition, it may be the case that participants in the current study never tried listening to music when learning a new skill; thus, this is not substantial evidence to suggest that music could not facilitate skill acquisition in golf [36]. Future experimental studies may consider investigating this area.

While qualitative data represent a small, nuanced sample limiting generalizability of results, the potential benefits from music use in golf as highlighted by the participants of the current study include the following: maintaining a desired swing tempo by matching the beat of the song to the tempo of their swing; improving attentional focus by distracting attention from unhelpful stimuli (e.g., superfluous swing thoughts, outside chatter) and maintaining focus on the task at hand (e.g., target, tempo); facilitating automaticity in the swing; achieving optimal physiological arousal (e.g., increasing energy or calming nerves); regulating mood, particularly in response to negative emotions following a poor shot or round; and increasing confidence, effort, motivation, task adherence, flow, and task enjoyment. In the next section, practical implications, limitations, and recommendations for future research are discussed in light of current findings.

## 6. Practical Implications

Golfers may find it particularly helpful to practice a desired swing tempo by using music similarly to the way one might use a metronome—to synchronize one’s movements with the beat. Matching the music to the golfer’s swing tempo is suggested. Furthermore, recalling a particular song in one’s head through auditory imagery may be a way to reap the benefits of music during competition, even when the golfer is not permitted to wear headphones. Auditory imagery to maintain swing tempo may be especially helpful during high-pressure situations, when a golfer’s swing is more likely to speed up due to increased nervousness or anxiety [37]. Music may also be a useful distraction for golfers who experience an internal attentional focus (e.g., excessive swing thoughts, negative self-talk) by occupying limited channel processing capacity and, thus, facilitating greater automaticity. This may be more appropriate for amateur and professional golfers who do not need to consciously focus on fine motor tasks or skill acquisition, as a beginner golfer would. 

Music may also play a valuable role in emotion regulation during practice and before competition by producing a calming effect for golfers who experience nervousness, or an energizing effect for those who tend to have lower levels of arousal. In golf, the goal is to feel relaxed, but not lethargic. Golf can be an incredibly frustrating and tedious sport, wherein emotional responses can translate into physical reactions such as increased bodily tension or a tighter grip on the golf club. In fact, researchers have found that the negative relationship between pressure and golf performance is often mediated by increased tension [38]. Music can be an effective tool in regulating emotion in sport [29]. Furthermore, maintaining a desirable mood state may produce other enhanced performance outcomes such as increased task adherence, effort, flow, and enjoyment, factors mentioned by participants in the current study. Channeling calming music or motivational lyrics may serve as a performance cue, allowing golfers to cope with negative emotions more gracefully (e.g., letting go of a poor shot) and encouraging positive emotions more conducive to performance (e.g., increasing confidence). Music may also be helpful during a golfer’s pre-performance routine to increase mental readiness or motivation.

Lastly, participants in the current study noted using 14 different music genres, which suggests that personal preference is diverse and may be a precursor to potential performance benefits. Interestingly, none of the participants reported a preference for listening to jazz music while playing golf, even though this was the genre that Baghurst et al. [2] found resulted in better putting performance compared to classical, country, rock, and hip-hop/rap. Given that athletes may exhibit differing degrees of optimal arousal and emotional states for peak performance [39], we stress the importance of music selection based on individual preference and tempo, as well as consideration of physiological, emotional, and motivational effects of such music. 

## 7. Limitations and Recommendations for Future Research

While beneficial in gaining insight into the specific areas of study interest, it is possible that semi-structured interviewing could have limited or missed aspects of participant experiences. Future qualitative research may benefit from integrating open-ended, non-directive interview approaches to gain additional depth and insight into participant experiences. Although the sample was equally represented in terms of gender, future studies would benefit from more diversity. For example, golfers in the current study ranged from 18 to 34 years old (*M* = 22.9 years). According to the National Golf Foundation [40], less than 5% of the 29 million total golfers in the United States (US) are under the age of 30. It is possible that music use may simply be a younger adult phenomenon; thus, future studies should include a representative sample to gain insight into the use and benefits of music with competitive golfers across a wider age range. 

It should also be noted that the intersection of contemporary performance-enhancing techniques with traditional golf rules and etiquette may constrain utilization in competitive contexts. Currently, the United States Golf Association (USGA) does not allow for the use of music “while making a stroke or for a prolonged period” as it “might assist the player in his play, for example, by eliminating distractions or promoting a good tempo” [41] (para. 1). This rule, however, states that a golfer may “listen to a device briefly … while walking between the putting green of one hole and the teeing ground of another hole” (para. 1) and also states “no restriction on listening to music … while practicing (whether on the practice ground or on the golf course, and whether by oneself or while playing with others)” (para. 2). The USGA notes that golfers should mind club rules and disciplinary codes in these cases. Investigation is needed to determine optimal music use during practice and its translation to competitive scenarios. Caution should be exercised when a golfer may exhibit dependence on music as a performance enhancer, especially if its use is prohibited.

## 8. Conclusions

Our preliminary qualitative findings suggest that the types and perceived functions of music use among golfers are aligned with the research in this area. Based on the findings of this study, further empirical investigation is needed to tease out the various related factors of music use in golf. It can be concluded that golfers in the current study found music to be a performance enhancer in various ways, either directly (e.g., increased effort) or indirectly (e.g., improved mood). Future research would benefit from quantitative studies examining the impact of music on variables such as golfers’ mood, arousal, performance, attention, motivation, and confidence.

Given the capacity of carefully selected music to elicit profound affective, neurophysiological, and behavioral responses [42], there is clear potential for golfers, coaches, and sport psychology practitioners to utilize music in working with golfers in a training context. In addition to potential benefits of music use in golf, it is also important to mention caveats and other considerations. Music should be used judiciously in order to avoid dependence during practice or performance. It should be treated as a tool rather than a necessity. Golfers, coaches, and mental performance consultants should also be mindful of the potential drawbacks of listening to music, such as violating golf etiquette or safety issues (e.g., not being able to hear a warning of “fore”). 

While our observations represent a small sample of amateur and semi-professional golfers, the growing number of golfers who use music in training and before competition warrants further investigation from both applied and empirical perspectives. Based on the findings of the current study, we encourage practitioners and researchers to design, test, and share the results of music-related interventions for golfers in future studies.

## Supplementary Materials

The following are available online at https://www.mdpi.com/2075-4663/7/3/60/s1, Table S1: Summary of Domains, Categories, and Core Ideas.
