# Supplementary material for: A Qualitative Investigation of Music Use among Amateur and Semi-Professional Golfers
Source: Sports (Basel). 2019 Mar 5;7(3):60. doi: 10.3390/sports7030060 (PMC6473220; doi:10.3390/sports7030060)
Supplement: Supplementary file 1 [file sports-07-00060-s001.pdf]

Table S1

Summary of Domains, Categories, and Core Ideas

Domain, Category, and Core Idea	Frequency
**TEMPO**	
Mental Synchronization Effects	General
Beat is helpful	
Play music in head when not listening to it	
Wrong tempo can disrupt performance, disturb focus	
Physical Synchronization Effects	Typical
Choose music for purpose of tempo control in swing	
Match swing to tempo	
Compare music to metronome	
**ATTENTION**	
Music Functions as an Associative Mechanism	General
Music functions as a focus mechanism	
Use music to concentrate on task	
Use music to narrow focus to helpful thoughts	
Music Functions as a Dissociative Mechanism	General
Use music as a distraction from other people	
Use music as a distraction from unhelpful thoughts	
Music Serves as a Memory Cue	Variant
Golf-related memories	
Non-golf-related memories	
**PHYSIOLOGICAL REGULATION**	
Music Helps to Regulate Energy/Arousal Level	General
Music helps to increase my energy if I'm too sluggish	
Music helps me to relax physically (calm nerves)	
Music helps regulate physical "feeling" (tension in body)	
Music Choice is Sport-Specific	Variant
Choose different music in golf vs. other sports	
Optimal physiological arousal level	
Music Facilitates Physical Automaticity	Variant
Use music to become automatic in movements	

**PSYCHOLOGICAL REGULATION**	
Music Helps to Regulate Mood	General
Music keeps me calm	
Music keeps me from getting too angry/frustrated	
Music puts me in a better mood (happy)	
I choose music based on my mood that day	
If a certain song comes on, it will change my mood	
Music Enhances Mental Performance State	General
Music puts me in a better psychological/mental state to play (mental preparation)	
Music helps me "bounce back" from poor performance (performance response)	
Music increases my confidence	
Music Increases Motivation	Typical
Lyrics are motivational	
**EFFECTS OF MUSIC ON PERFORMANCE PERCEPTIONS**	
Music Affects Time Perception	General
Time goes faster when I listen to music	
Music Functions as a Performance Enhancer and Facilitates Flow	General
I am more efficient when listening to music	
The task seems easier when listening to music	
Music directly enhances my performance	
Music increases effort level	
Music facilitates flow state, gets me "in the zone"	
Music Improves the Qualitative Experience of the Task	Typical
Music makes golf more enjoyable	
Music prevents me from getting bored	
**TO USE OR NOT TO USE**	
Social/Individual – Music is used with other people and when practicing alone	General
Setting – Listening to music is common during pre-performance routine and practice	General
Difficulty – Music may be unhelpful when learning new skills	Typical
Task – Music is used more often in putting vs. driving	Typical
Music is not commonly used in conjunction with imagery	Typical

Note. General indicates responses from 9–10 participants (90–100%), Typical indicates responses from 6–8 participants (60–80%), and Variant responses from 2–5 participants (20–50%).
